# Beyond the Chest Wall: A Case of Fungating Lung Tumor and Review of Literature

**DOI:** 10.7759/cureus.87741

**Published:** 2025-07-11

**Authors:** Jad Kabbara, Tala Mobayed, Moutafa Moussally

**Affiliations:** 1 Anesthesiology, Lake Erie College of Osteopathic Medicine, Westlake, USA; 2 Internal Medicine, American University of Beirut Medical Center, Beirut, LBN; 3 Surgery, American University of Beirut Medical Center, Beirut, LBN

**Keywords:** complex surgical resection, fungating tumor, lung cancer, lung surgery, rare cancers

## Abstract

Lung cancer is the leading cause of mortality among cancers. Fungating tumors are rare manifestations of advanced lung cancer in which the tumor invades the chest wall and breaks through the skin. These tumors can have devastating physiological and psychological effects on patients. They confer poor prognosis to the affected patients. Furthermore, symptoms such as bleeding or foul smelling exudative discharge are associated with fungating tumors. When deemed suitable, surgical resection with chest wall reconstruction is the treatment of choice. We hereby present the case of a lung tumor invading the chest wall with extension to the skin. We also perform a review of literature on fungating tumors.

## Introduction

Lung cancer is the driving cause of cancer death in both sexes and stands as the second most common malignancy in the world, with roughly 20% of diagnosed cancers being of pulmonary origin [1]. Most patients present with an advanced stage of illness, highlighting the aggressivity of the disease [2]. Moreover, symptoms can range from local tumor effects such as cough to metastatic disease effects such as back pain [3]. A very rare presentation is a fungating tumor, which is a malignant mass that breaks through the skin and disrupts its integrity in locally advanced or metastatic cancers, which occurs in 5%-14% of patients with advanced or metastatic cancer [4]. Fungating tumors are ulcerated, necrotic malignant masses that are prone to infection and bleeding, making them very distressing for both patients and caregivers. We hereby present the case of a fungating lung tumor and perform a review of literature on fungating tumors.

## Case presentation

Our patient is a 79-year-old male patient with 60 pack years smoking history who presented to our emergency department for a bleeding chest ulcer. The patient’s medical history is significant for hypertension and dyslipidemia. He reports having discovered a bump on his chest six months ago that has progressively enlarged since then. The patient reported that the bump was painless and asymptomatic, which prompted him not to seek any medical advice. However, he noted that the lesion had become malodorous in recent weeks. He also reported that he started having mild bleeding from the lesion, which became profuse on the day of presentation. The patient denied any fevers or chills. He reported significant weight loss and decreased appetite. The patient did not complain of any dyspnea, chest pain or cough.

**Figure 1:**
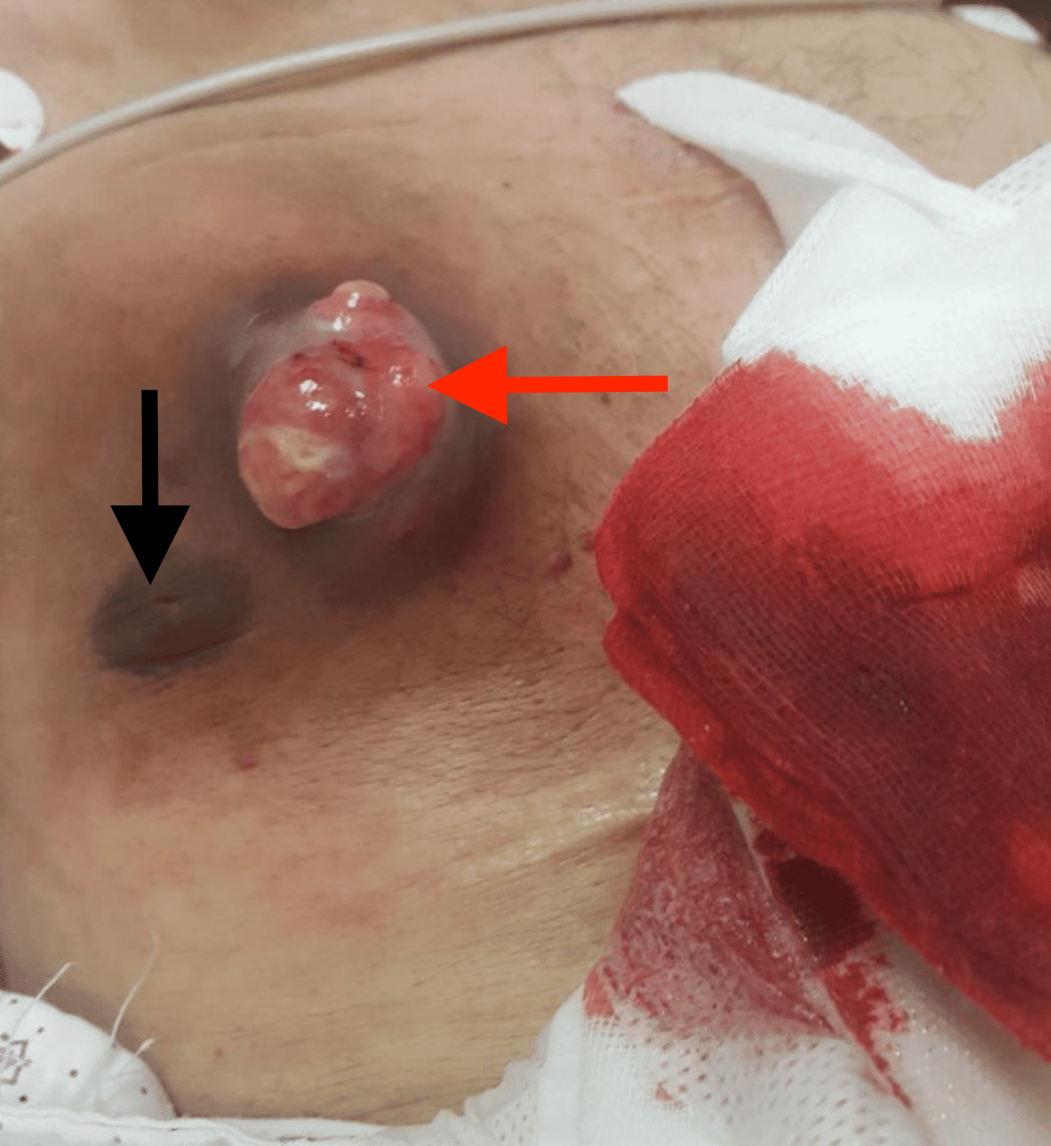
Bleeding fungating tumor along right chest wall of patient upon presentation (black arrow representing the nipple) (red arrow showing the tumor)

On physical examination, the patient was found to have an approximately 6 cm*6 cm bleeding lesion on his right anterior chest (Figure 1). The lesion was non-tender, firm and immobile. The overlying skin was ulcerated and oozing. Laboratory findings were as follows: hemoglobin, 7.5 g/dL (13.5-17.5 g/dL); hematocrit, 22% (41%-53%); platelets 144,000/mm^3^ (150,000-450,000/mm^3^); white blood count 6800/mm^3^ (4000-11,000/mm^3^).

**Figure 2:**
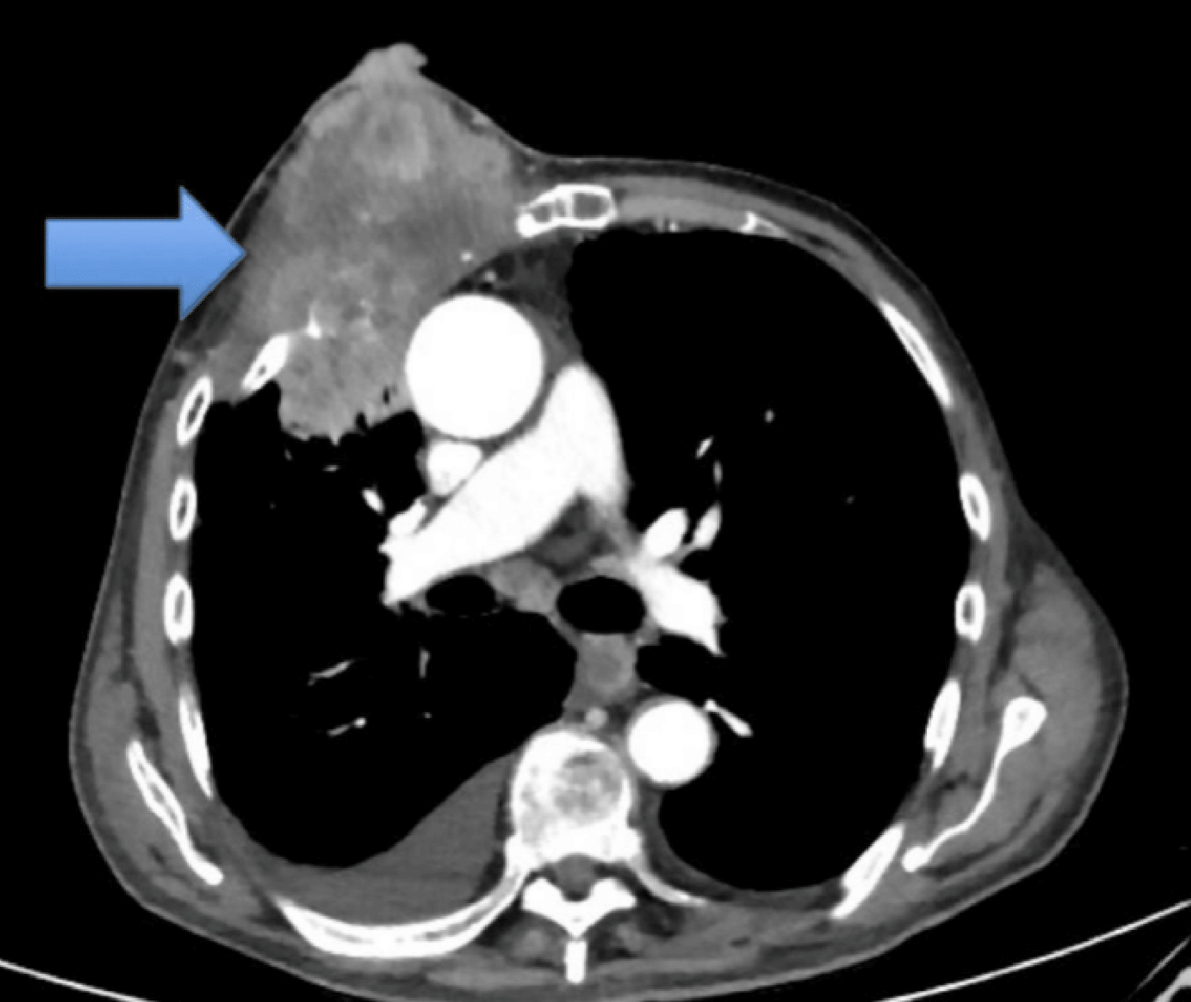
Contrast-enhanced computed tomography (CT) image showing right chest wall mass measuring 11x6 cm invading the third and fourth rib and extending superficially towards the skin

Creatinine and liver function panel were within normal range. Contrast-enhanced computed tomography of the chest was done and revealed a multifocal right chest wall mass measuring 11x6 cm (Figure 2). The mass is centered in the right second and third intercostal spaces anteriorly, with the destruction of the anterior aspects of the right third and fourth ribs. The mass has superficial extension with invasion of the skin, ulceration and extrusion externally. The patient then underwent tissue biopsy, which revealed high-grade poorly differentiated non-small cell lung carcinoma. Immunohistochemical staining was positive for TTF-1 and negative for p40, supporting a diagnosis consistent with adenocarcinoma subtype. Based on imaging and clinical findings, the cancer was staged as T3N2M0 at the time of diagnosis. The patient refused any medical intervention and opted for palliative care instead. Palliative care focused on symptom relief, comfort, and quality of life, with a heavy emphasis on psychosocial support.

## Discussion

The patient was diagnosed with poorly differentiated non-small cell lung carcinoma, which usually presents with symptoms of cough, hemoptysis, weight loss, and chest pain. However, this case is extraordinary for its unusual presentation as a fungating tumor, a rare lung cancer that breaks through the skin, invading the surrounding tissue.

The diagnostic workup of lung cancer must include both radiologic imaging and histopathological examination for proper diagnosis and staging [5]. Differentiating between small cell and non-small cell lung cancer is essential [6]. Fungating lung tumors are usually considered T3 or more. Early workup should include computed tomography (CT) scan of the chest or magnetic resonance imaging (MRI) in some cases followed by tissue biopsy [7]. Bone scintigraphy and cranial MRI can also be performed to assess metastasis. Positron emission tomography (PET)/PET CT is capable of determining tumor stage, especially in small cell lung cancer [6]. Unfortunately, people detected with advanced disease suffer from remarkable and distressing symptoms, especially if the malignant lesions presented as fungating or ulcerating masses [8].

These invading masses usually result from a present tumor rapidly swelling and increasing in size, manifesting the progression of the disease and placing the patient in a worrisome state [5]. Unfortunately, patients presenting with such masses are considered to have poor prognosis due to a low healing rate [4]. Development of such malignant wounds usually occur in the last six months of cancer [9]. Moreover, it is calculated that these masses appear in only 5% to 10% of people with all cancers with a very rare chance of healing [10].

Fungating masses pose a strenuous task for physicians and the patient him/herself. They can be associated with a foul-smelling odor, bleeding, pruritus or exudate [11]. The patient can also present with other symptoms such as dysphonia due to compression of adjacent structures by the enlarging lung mass [5]. Initially, the tumor invades the parietal pleura, continuing into the soft tissue and progressively into the intercostal muscles, finally making it through the ribs and penetrating the skin. Moreover, chest pain will be the dominant symptom. Hemoptysis and dyspnea can be present too. The tumor’s infiltration and extension displays itself clinically as weight loss, hypoxemia and digital clubbing [7]. Functional impairment is present if the wound is open along with microbial contamination and necrosis, resulting in a malodorous pus [4]. Necrosis occurs due to tissue hypoxia when the mass interferes with tissue oxygenation and hemostasis [10].

Care for wounds resulting from fungating tumors may be challenging [11]. Nevertheless, healthcare providers should always establish goals for proper wound management and healing along with symptomatic management strategies [12]. The ulcerating tumor’s size, location and shape can help establish the goal of treatment. The application of wound dressings helps alleviate odors, decrease the amount of bleeding, and absorb exudates. Moreover, applying a dressing covers the disfigurement and brings relief to the patient. It is important to keep the wound surface moist to prevent adherence and to apply topical antibiotics to reduce microbial growth and hence eradicate bad odors [4]. It is also imperative to realize that simple measures such as cleaning the wound daily with soap and water along with light debridement can be helpful [12].

Tumor necrosis can be difficult to handle for both caregivers and patients. If necrosis is present, debridement may be suitable. However, the choice to perform debridement should weigh the risks and benefits [4]. Necrotic tissues are an ideal medium for anaerobic bacteria, highlighting the importance of antibiotics in this setting. Although a wide variety of antibiotics target anaerobic bacteria, metronidazole is the one mostly used [13]. Moreover, bleeding ulcerating wounds can also be present. Local care to bleeding fungating tumors is crucial since the majority of the wounds present with minor bleeding either spontaneously or after a dressing change. Minor bleeding can be controlled by topical epinephrine, while major hemorrhages are better contained by surgical sponges or by the use of topical or systemic tranexamic acid to help establish homeostasis [4]. Interventional techniques such as angiography or endovascular embolization are effective against massive bleeding [11]. When a lesion fails to respond to proper handling, patients become hopeless and overwhelmed with apathy, leaning to palliative care and pain control [8]. Chemotherapy or radiation therapy are palliative oncologic approaches that, if possible, can succeed in reducing tumor burden [11].

Locally advanced chest wall tumors invading the skin can be managed surgically. This can be done via surgical resection with appropriate flap or graft reconstruction. Nonetheless, the decision to perform any surgical intervention must be individualized and entail meticulous analysis of the risks and potential benefits of surgery in the context of the overall disease status [14]. In case of tumor invasion to paravertebral sulcus, disarticulation of the rib from the vertebra is required. Lobectomy is usually the next step with lymph node excision [7]. Intraoperative frozen specimens of soft tissue are not beneficial [15]. Reconstruction with prosthetic material of the chest wall is instrumental for anatomical maintenance and protection of vital organs while preserving ventilatory mechanisms [7]. In some cases, reconstruction is not needed if the defect is small (<5 cm) and less than three ribs are resected. Reconstruction of anterior chest wall defects should be done with vigilance due to the proximity to the heart [15]. Complications of chest wall reconstruction include local infection and a mortality rate of 6 percent post op due to respiratory failure [7].

## Conclusions

Fungating masses comprise rare presentations of lung malignancies. These masses, which usually indicate an advanced disease, are life-threatening and pose huge burdens on both patients and caregivers. After a complete medical assessment and confirmation of the diagnosis, medical intervention should be initiated, taking into consideration the patient’s individualized choice of treatment. Fungating tumors not only cause physical symptoms but also carry with them psychological suffering due to the disfigurement caused by their appearance. Determining the nature of the lesion, whether infected, necrotic, or bleeding, is essential for wound care protocols as well as adjunctive therapies like surgical intervention or antibiotics. Radiation therapy can be highly effective in reducing the size of the lesion as well as assist in bleeding and pain relief, especially in those who are not surgical candidates. Thus, these lesions should be cared for meticulously by multidisciplinary teams, including radiation oncology, surgery, palliative care, and wound care specialists, allowing for a holistic patient-centered management plan that ultimately focuses on symptom relief and disease control.
